# Harris’s Principles and Practice of Dental Surgery

**Published:** 1848-04

**Authors:** 


### HARRIS'S PRINCIPLES AND PRACTICE OF DENTAL SURGERY.

The third edition of this valuable work is just from the press of Lindsay & Blakisten, Philadelphia. The work has too long been in the hands of the profession, not only in America, but in Europe, to require any particular notice commendatory or otherwise. We presume that no work on the same subject from the American press has ever met with so extensive a sale; and if we recollect aright, it is the only work which has passed to its third edition. The first chapter is devoted to the rise and progress of Dental Science in Europe and the United States. The second, to "the importance attached to the human Teeth, and the customs concerning them." We have thought the size of the work would justify the leaving out of these two chapters; particularly as most of our other works (unless indeed it be pocket manuals,) contain much the same matter.

The remainder of the work is divided into seven parts:

Part 1st—Anatomy and Philosophy of the Mouth. This embraces only that part what might be strictly called dental anatomy, and without a knowledge of which no man should attempt operations on the mouth. Some excellent plates are interspersed through this part of the work—and will aid the student very much in this part of his studies.

The 2d part is appropriated to the physical condition of the Teeth, Gums, Salivary, Calculus, &c. This is an important study, and when well understood enables the student or practical dentist to take up and the more readily appreciate

The 3d part of the work, which is devoted to the diseases of the Teeth and their treatment. This embraces the cause and prevention of caries,—filing, filling, extracting, cleansing,—description of the instruments used, &c., &c. Here, also, we have plates representing the various forms of forceps used in extracting,—and particularly Dr. Maynard's improved drill. This we should think a valuable instrument. The elevator, (*c*) figure 72, we use a great deal for the extraction of front roots, and indeed for the roots of almost all the upper Teeth. We have the blade made so thin that with a little effort it can be forced between the alveolar process and root; then using the process as the fulcrum of the elevator, giving, when the roots are round, rather a rotary motion, they usually yield to the power thus exerted without any difficulty. Yet when there is strength of root sufficient for the forcep above the alveolar border—we almost invariably use the forcep.

Part 4th is devoted to the diseases of the Gums and alveolar processes—salivary calculus, &c. We must confess this is a part of the science with which the majority of the profession appear to be but little acquainted. We commend this part of the work to the especial attention of such. It is just as important to save a sound as diseased tooth.

Part 5th treats of diseases of the maxillary sinus. This is a subject with which every dentist should be conversant,—for although comparitively rare in its occurrence—yet he knows not what day or hour he may be called on to treat some form or other of disease which attacks this sinus. Besides, such cases are often thrown on his hands by physicians, and his skill and judgment is thus brought in contact with those who have made general medicine their study. A due regard for the honor of the profession should induce every dentist to be prepared for such an occasion, that he may make the importance of our science felt and duly appreciated. The author has collected the views of the most prominent writers on this subject, and in addition given many cases and much valuable information.

Part 6th.—This is taken up with mechanical dentistry, and presents a fair and almost complete state of this department at the present time. We are glad to notice the continued improvements almost daily made in this part of our profession. Those of us who can look back twenty years and compare the then and present condition of mechanical dentistry, can best appreciate these improvements. Many inventions, it is true, prove useless, except, indeed, that they lead to that which may be of utility. But a work could scarcely be so complete and perfect in this department of our science, but that the same author would at the end of a year make more or less additions and alterations. We gave in the last number of the Register a recipe for an adhesive wax—which, simple as it is—will enable the dentist to arrive at a neatness and accuracy in the articulation of his teeth that cannot be obtained by any articulating model. We first take up our articulating casts as recommended by Dr. Harris—for we have never seen any instrument equal to the plaster articulation. But after we have gone thus far, and have our teeth ground in to fit, we fasten them to our plates with this wax, and attach our springs, arranged as we wish them in the mouth. We then have our patient come in that we may try them in the mouth—and the wax will hold the teeth sufficiently firm to allow free use of the jaws and considerable pressure on the teeth. If we find a tooth not set as we desire, it is removed and adjusted to fit. In this way we have repeatedly inserted full sets, and not suffered the grinding surface of a single tooth to be touched with the emery wheel. We also feel a certainty that no alteration will have to be made after the teeth have been soldered to the plate. The same course is most usually pursued in partial sets.

Part 7th is exclusively devoted to diseases and defects of the palatine organs, and the remedy. There is, perhaps, nothing in the line of our profession more trying to the patience and skill of the dentist than cases of this nature which are sometimes presented for remedy.

Many cases, it is true, present no difficulty to the eye or skill of the ingenious dentist—requiring but little more than a plate to cover the opening, and clasps to fasten the same to the teeth. Indeed, we do not consider the addition of a few teeth to the plate as generally much increasing the difficulty of the operation. But when the soft palate is involved in the disease, and the veleem and uerela are also to be restored—difficulty after difficulty presents itself, and but few cases of this kind would justify the labor and expense to even make it of (as we think, as regards comfort and permanency,) doubtful utility. This part of the work, however, contains some excellent plates; and what is perhaps better, sound and correct pathological views, which should never be lost sight of in the treatment of injuries resulting from diseases of the palate.

We have hastily run over the arrangement of the work, so that the readers of the Register can form an estimate of its contents. The division as adopted by the author we think as convenient and advantageous. Indeed, we regard the work as one which should be in the hands of every dentist.

We have not intended anything like a review of the work—for our time and other duties would not permit this, if we thought it necessary.

We would be glad, however, to draw the attention of the profession to this as well as many other works which we may hereafter allude to in the pages of the Register. We consider dentistry as a science too far advanced now, to allow those who practice it as a profession to feel that after having read one or even two volumes on the science, that they had all that is requisite, or indeed possess more than the beginning of a library. We, however, have met with those, and that lately, who, after having read not more than one author on the subject, (and that very imperfectly,) assume all the knowledge from Hunter to the present time, and hint at many great discoveries and improvements made,—thus eclipsing all that had ever been heard or read of in the profession. Young sprouts, are they, germinated by the touch of some magnetic influence, and developed and matured by telegraphic wires. The old beachen rod never came across their backs.

